# The Power Elite in Greenland

**DOI:** 10.1111/1468-4446.70002

**Published:** 2025-06-04

**Authors:** Morten Fischer Sivertsen, Anton Grau Larsen, Christoph Houman Ellersgaard

**Affiliations:** ^1^ Department of Business Humanities and Law Copenhagen Business School Frederiksberg Denmark; ^2^ Department of Social Sciences and Business Roskilde University Roskilde Denmark; ^3^ Department of Organization Copenhagen Business School Frederiksberg Denmark

**Keywords:** elite networks, elites, greenland, island societies, postcolonialism

## Abstract

In this research note, we map the power elite in Greenland, amidst the current geopolitical interest in the nation. Using social network analysis, we identify a power elite of 123 individuals as the central circle in an extensive affiliation network data on 3412 positions held by a total 2052 individuals in 456 affiliations. We find an integrated and cohesive power elite dominated by actors from politics and public and private enterprises. When comparing this central circle to the previous studies of power elites in the former colonial power and current sovereign, Denmark, the political sector and the state are stronger in Greenland at the expense of the private sector. However, while the elite is integrated, we also identify potentials of fracturing. Thus we find a division between politicians—who are more likely to have childhood and educational ties to Greenland—and other elite groups—in particular private business—who are more likely to have academic degrees, be male and live in the Capital, Nuuk. The network of the elite is also clearly clustered around the strength of affiliation with Greenlandic society. We conclude by discussing how the potential fracturing of the Greenlandic elite along ethnic division lines may lead to a lack of cohesion and legitimacy entering the current geopolitical tensions surrounding the world's largest island.

## 1 Introduction: Big Decisions Looming for the Small Elite on the World's Largest Island

‘I think we're going to have it’, President Donald Trump told reporters on Air Force One on 25 January 2025, doubling down on his desire to buy the island from the Kingdom of Denmark first proposed in 2019. In the weeks before his inauguration, acquiring Greenland became a key means to deliver expanded US territory and reinforced geopolitical strength, culminating with Trump sending his son, Donald Trump Jr., to visit the island. Thus the geopolitical struggle over hegemony of the island's 57,000 inhabitants and more than 2,000,000 square kilometres—more than 50 times the territory of Denmark—is on. The former colonisers of Greenland, the Danish state have been placing their bets on the will of the people of Greenland with Prime Minister Mette Frederiksen asserting that ‘Greenland belongs to the Greenlanders’, thus putting the determination of Greenland in the hands of the island's population.^1^ After the March 2025 elections in Greenland, which saw significant gains for both the centre‐right, gradual‐independence party Demokraatit and the pro‐immediate independence party Naleraq, the battle for the hearts and minds of the Greenlandic people is unfolding. This is also reflected in renewed signs of strong interest from the U.S. administration—most recently with a visit from Vice President J.D. Vance just weeks after the election. Key to this struggle over the future alignment of Greenland is the tiny group of people making key decisions in the country, the Greenlandic power elite. The positioning of this elite could play a key role in deciding an outcome that could shape transatlantic relations in the future.

In this research note, we present our analysis of the composition of this power elite. Using social network analysis, we use formal affiliations to identify a core of 123 individuals. By looking at their sectoral affiliation, educational background and ties to the greenlandic society, combining with qualitative interviews, we assess the interests of these key actors in continuation of relation with the colonial power of Denmark versus entering US dominion. Understanding these dynamics is crucial for assessing whether power in Greenland is concentrated within a small elite or more broadly distributed, and the degree to which such power resides among Greenlandic actors or remains in the hands of Danish elites with historical, cultural and institutional ties to Greenland. By mapping these structures, we contribute to a deeper understanding of Greenland's democratic landscape at a time when its political future is being intensely debated. Thus we ask the following research question: *Which sectors dominate formal elite networks in Greenland and what does the demographcis to this elite tell about the power structure, and its cohesion, in Greenland*? Our results suggest that a substantial part of the power elite in Greenland has strong links to Danish society. However, we also find a divide between those who grew up on the island and thus are likely to have strong embedness with the indigenous population vis‐a‐vis those with stronger links to Denmark. This could potentially cause a rift in the Greenlandic power elite in which anti‐colonial sentiment could lead to a fraction of the political elite going against the general interest of the power elite and use the American overtures as an opportunity to leave, akin to the Brexit movement in the UK.

## 2 Background: Power Elites in Postcolonial Island States

A part of the Kingdom of Denmark, Kalaallit Nunaat (Greenland) has some degree of sovereignty regarding domestic government, with some areas under local jurisdiction and others—not least matters that relate to foreign policy and security—still managed from Copenhagen (Grydehøj 2020). On an index of formal sovereignty ranging from 0 to 1 for island territories, Greenland scores 0.42 because of their only partial diplomatic, judiciary and legislative sovereignty and lack of diplomatic and monetary sovereignty (Alberti and Goujon 2019).^2^ The 2009 Self‐Government Act states, however, that Greenland can gain independence if a political majority, a public referendum, and approval from the Danish Parliament support it. Greenland, with its vast territory and scattered settlements, shares geographic similarities with Pacific island nations like Kiribati. However, unlike these nations, Greenland remains closely tied to its former colonial power, Denmark, which has historically influenced its language and culture through ‘welfare colonialism’ (Connell 2016).

To understand Greenland's power structure we draw on C. Wright Mills (1956; 1958) concept of the power elite, which describes how a small, interconnected circle of individuals dominates key institutions and decision‐making processes. Mills' work has since been continued in the power structure research approach (Domhoff 2007), using formal networks to analyse cohesive groups—but also fracturing and therefore lack of political cohesion (Mizruchi 2013)—within elites. However, microstates such as Greenland are characterised by both a unique form of public administration based on the social proximity between senior public servants and the population (see Baker 1992) and ‘overlapping role‐relationships’ (Benedict 2004), cross sectoral and interdependent networks where each individual plays several roles (Ravn‐Højgaard 2022), as also described in Barnes (1954) seminal study of the communities in the Norwegian island parish of Bremnes. Nonetheless, looking at formal political networks offers a way to assess how power relations—even if they are based on informal connections—have become formalised.

Previous mappings on the elite using a positional sample in Greenland have shown an elite with strong ties to Denmark (Christiansen and Togeby 2003), a clear trend towards ‘Greenlandization’ between 2000 and 2009 is seen by more people with either educational background in Greenland or no career positions in Denmark taking up elite positions, in particular in public administration and state owned enterprises, while private businesses remained controlled by individuals with a Danish background (Ankersen and Christiansen 2013). These studies show that there is ‘strong cohesion between elite groups’ based on elite individuals having positions across sectors, but do not formally analyse the links between groups as we do identify power elite and its cohesion and potential fracturing.

## 3 Methods and Data: Mapping the Central Circle

To identify the overlapping circles of the power structure in Greenland, we have collected data on all potentially powerful affiliations in Greenland with a formal membership list. Our dataset consists of 3412 positions held by 2052 individuals in 456 affiliations within private and public enterprises, the civil service, civil society organisations, industrial relations unions, and the academic world, collected between February 1, 2019, and February 1, 2020. The primary data sources included publicly accessible online resources such as the Danish Central Business Register, the website of the Greenlandic government, and the websites of political parties, municipalities, and interest organisations. A significant portion of decision‐making power still resides in Denmark because these policy areas are governed from Copenhagen. While politicians and civil servants in Copenhagen are governing policy areas still under Danish responsibility of course making decisions having important consequences for the population in Greenland, they are embedded in the Danish and not the Greenlandic power structure. Therefore we have not included governing bodies covering the entire Kingdom of Denmark in our dataset, but also those based in Greenland. Furthermore, a key limitation of this data is its inability to capture informal power structures, which play a significant role in small states (see above). Lastly, we acknowledge that important developments may have occurred since we collected our data in 2019–2020. For instance, we note how some commentators have pointed to an increasing number of Greenlanders entering into top positions within the state, probably as a result of a growing share obtaining their degrees from the University of Greenland, who launched a bachelor's programme in Law in 2018.^3^ At the same time, however, several studies (Ellersgaard and Larsen 2023) point to a relative stability in elite networks over time. With this limitation in mind, our data still serves as a useful basis for understanding the structure of elite networks of Greenland.

**FIGURE 1:**
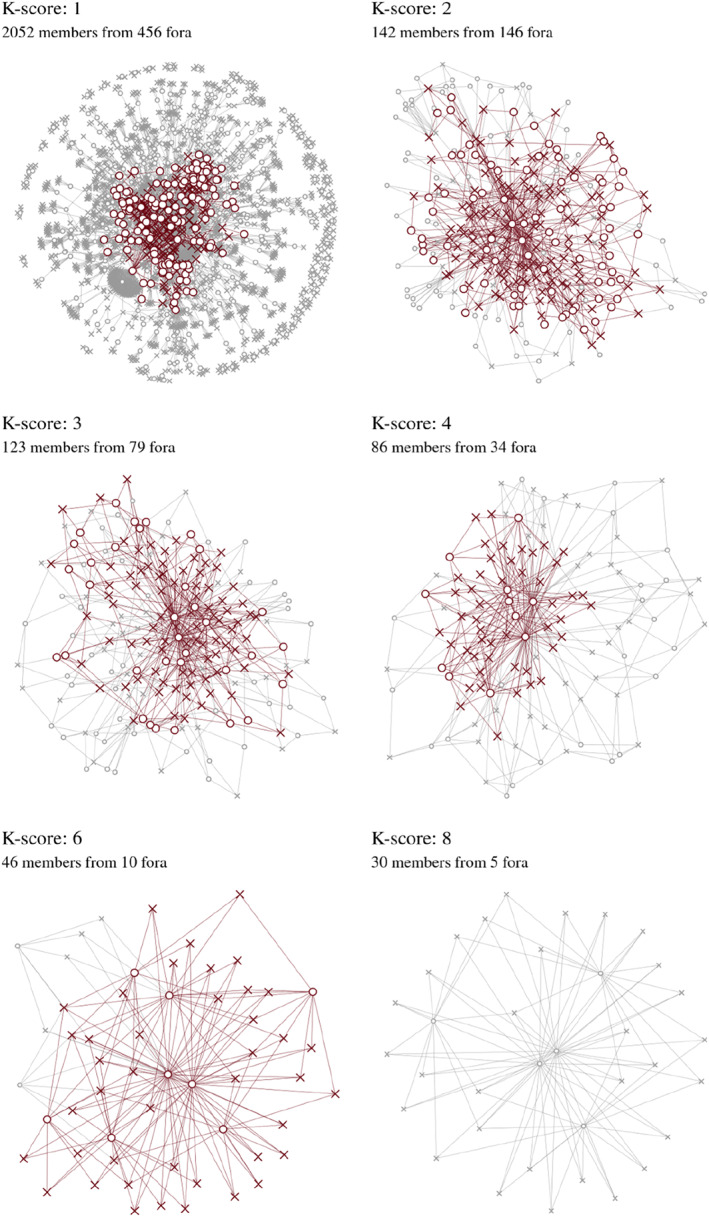
Individuals and affiliations removed by the iterate *k*‐circle analysis (in grey) used in identifying the central circles in the greenlandic elite. A *k*‐score of 3 was set as the threshold. x denotes individuals, o denotes affiliations.

To identify the core of this network, we employed the *k*‐circle analysis method, a variant of *k*‐core analysis (Larsen and Ellersgaard 2017). This approach identifies the most central social circles within an affiliation network and assigns each individual and forum a value based on their proximity to these circles. The higher the value, the closer the individual or forum is to the most connected part of the network, thereby playing a more critical role in binding the network together.^4^ Using this approach, we can identify different degrees of integration in the two‐mode networks in Greenland, see Figure 1. Based on previous studies which have identified elites of 115 individuals in 2000 and 127 in 2009 using a positional approach (Ankersen and Christiansen 2013), we select the *K*‐circle score of three, identifying a core of 123 individuals with at least two positions in the 79 affiliations that also at least hold three other of these individuals. We then collect prosopographical data on these 123 individuals, including their primary affiliation, gender, place of birth, place and type of education, residence and an assessment of the duration of their link to Greenland. Here we distinguish between those born in or having completed any level of education in Greenland, interpreted as being with Greenlandic childhood ties (72, 59%), all others (48, 39%)—of which almost all have Danish origins—and those we could not obtain information on (3, 2%). In the present political situation, having links to Greenland going back to one's childhood could be of significance for how one can be positioned in the debates on independence and potential realignment towards the US. Therefore, we explore the extent to which the network and sectors within the network are composed with regard to Greenlandic background.

This method allows us to compare the composition of the power elite in Greenland with that of Denmark, which was identified through similar types of data and roughly similar methods in 2017 and 2012 (Ellersgaard and Larsen 2023).

Lastly, we have conducted 6 qualitative interviews with key individuals in the power elite to get their perspective on the results of the network analysis in particular and power dynamics in general.

## 4 Analysis: The Structure of Power in Greenland

**FIGURE 2:**
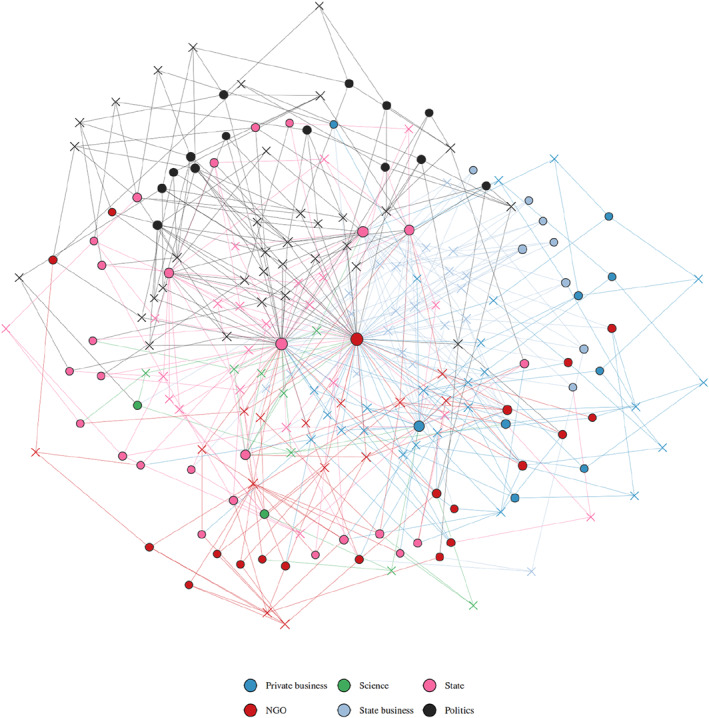
The network of the power elite in greenland. x denotes individuals, o denotes affiliations, node size is degree in the two‐mode network.

The overall conclusion of identifying the power elite in Greenland through social network analysis is that we do find a cohesive group in the core of these elite networks, primarily drawing on political and state power to obtain their position, see Figures 2 and 4. Looking at the sectoral composition, see Figure 2, of this group, it is dominated by politicians, who are 34 of the 123 individuals (28%). The politicians are primarily members of the Greenlandic government, Naalakkersuisut, and parliament, Inatsisartut, but also city mayors and members of the governing committees of the key political parties. The 22 (18%) senior civil servants compose around a fifth of this group, as do the are 24 (20%) private business leaders and 21 (17%) executives from state owned enterprises. In Denmark 43% of the 2017 power elite were business leaders in the private sector with less than a handful associated with state owned enterprises. Furthermore in Denmark, politicians and senior civil servants only constitute 10% each (Ellersgaard and Larsen 2023).

Completing the circles of power in Greenland are 14 leaders (11%) from interest organisations, including unions and business associations and 8 individuals (7%) tied to science and education, including university presidents and professors who are members of the economic council. Both these groups are less prominent than in the Danish power elite, where union leaders and business association executives constitute 25% of the 396 in the core of the network in 2017 and individuals from the academic sector hold 10% of the positions. This points to a situation in Greenland where, compared to Denmark, elite networks are not dominated by corporate interest but rather the political and state elites including leaders in the state owned businesses.

**FIGURE 3:**
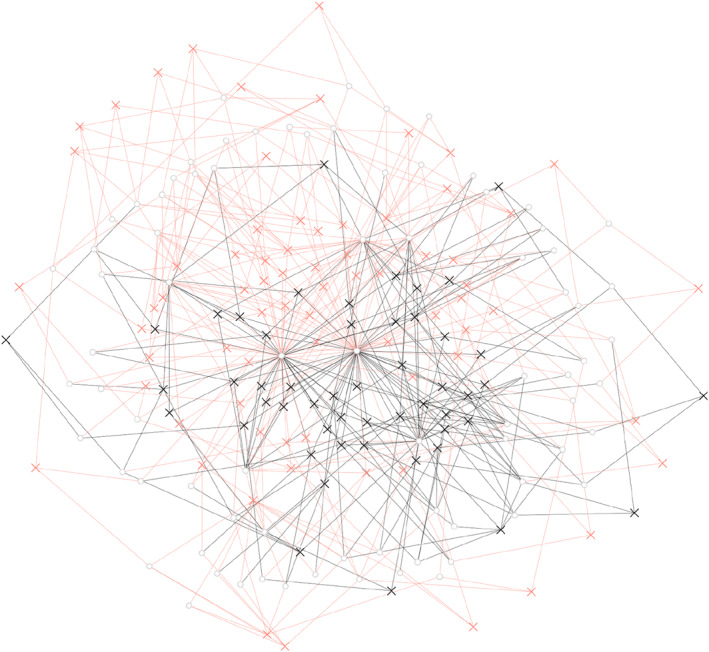
The individuals in the greenlandic power elite, by greenlandic background (childhood or educational ties to greenland, orange) or not (no pre‐adulthood Greenlandic ties, black). x denotes individuals, o denotes affiliations.

While we find integration along these sectors in Greenland, the analysis also points to potential fracturing lines in the power structure in Greenland. From Figure 2, we can also observe some degree of clustering separating private business, state and politician in three overlapping, but also distinct groups. Adding to this, in Figure 3, a substantial clustering of those with ties to Greenland from their education or childhood as opposed to those with a Danish background, particularly in the upper left corners of Figures 2 and 3, where the politicians are clustered.

**FIGURE 4:**
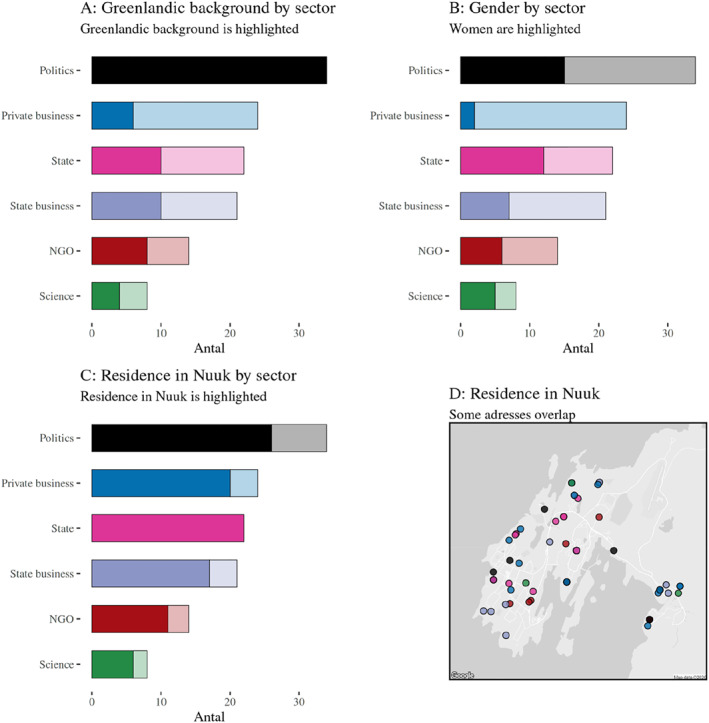
Key demographics for the greenlandic power elite by sector.

Furthermore, we also see this demographic difference between sectors. The political elite is unanimously tied to Greenland, whereas the other elites have around half who do not have any childhood or educational links to Greenland, with the private sector being clearly dominated by those without formative connection to Greenland, see Figure 4 panel A. Our qualitative interviews suggest that this placed the bureaucrats as key linkers between the all‐Greenlandic politicians and the Danish private sector.

The potential fracturing lines between sectors are extended by differences in professional background, see Table 1. Only 5 of 34 politicians (15%) have advanced educational degrees in Economy, Law and Administration or Engineering and Natural Sciences, compared to 53 (60%) of the other elite members. Instead, among politicians we find a majority with a background in welfare professional or vocational education. Compared to the Danish power elite (Ellersgaard and Larsen 2023), fewer power elite members have a background in traditional elite professions of Economy, Law and Administration—41% compared to 59% in Denmark—and Engineering and Natural Sciences—6% compared to 14% in Denmark—showing stronger similarities between Danish elites and business and state elites in Greenland.

**TABLE 1:** Educational background of the power elite in Greenland by sector.

Education							
Sector	Economy, law and administration	Engineering and natural sciences	Humanities	Welfare professions	Vocational	Unknown	Total
Politics	5 (15%)	0 (0%)	4 (12%)	7 (21%)	13 (38%)	5 (15%)	34 (100%)
Private business	13 (54%)	2 (8%)	0 (0%)	0 (0%)	4 (17%)	5 (21%)	24 (100%)
State	12 (55%)	2 (9%)	4 (18%)	1 (5%)	0 (0%)	3 (14%)	22 (100%)
State business	13 (62%)	2 (10%)	2 (10%)	0 (0%)	2 (10%)	2 (10%)	21 (100%)
NGO	4 (29%)	0 (0%)	2 (14%)	1 (7%)	2 (14%)	5 (36%)	14 (100%)
Science	4 (50%)	1 (12%)	2 (25%)	0 (0%)	0 (0%)	1 (12%)	8 (100%)
Total	51 (41%)	7 (6%)	14 (11%)	9 (7%)	21 (17%)	21 (17%)	123 (100%)

In the entire power elite, we find 47 women (38%)—a substantially higher proportion than in Denmark (26%). However, these women are primarily found in politics and state administration and are still marginalised in the private sector, where only 10%—two of 22—are women. This suggests very different, gendered recruitment patterns across elite groups. Similarly, we find that it is mostly politicians who manage to be part of the power elite and live outside of the capital, Nuuk (on the role of Nuuk as both centre and periphery, see Grydehøj 2014), see Figure 4 panel C. However, unlike in Denmark, where the power elite is somewhat segregated in their choice of residence with particular fractions living in different types of affluent areas, the Greenlandic power elite is somewhat scattered all over Nuuk (Panel D).

## 5 Concluding Discussion: Cohesive Elite, but Potential for Fracturing Along the Greenlandic‐Danish Divide

In this research note, we have tried to show the potential for mapping elite structures in postcolonial societies using social network analysis of formal affiliations. While this approach allows us to identify a core group within the network, it is not unproblematic. In a small society with less than 60,000 inhabitants, the formal ties we map here are underpinned by informal relations, kinship ties and a strong local embeddedness. However, mapping the formal ties allow us to identify how the different organisations in Greenland have organised their exercise of power and to which groups are involved in this organisation. With the Greenlandic society suddenly entering the whirlpool of global geopolitics, this organisation, cohesion and the potential lines of fracturing within are key to understanding how the Greenlandic elite may respond to the American propositions.

Compared to other elite groups, the power elite in Greenland is—while integrated through formal networks—much less homogeneous. The political elite and those with ties to the state through the bureaucracy or state owned enterprise, dominate, unlike in Denmark, where the economic elite is more dominant. While six of ten in the Greenlandic power elite as a whole grew up in or were educated in Greenland, signifying strong community ties—this is the case for all politicians. Thus, there is a potential for elite fracturing based on ethnic divisions, which our interviews also confirmed. Also, these may be further strengthened by differences in educational levels and gender composition of the different elite groups.

While we find a cohesive network in the Greenlandic power structure, we also identify potential internal cleavages associated with the ability to claim a Greenlandic identity, fracturing the political elite against other elite sectors, not least private business and state. For instance, in our qualitative interviews, senior civil servants frame politicians as ‘opportunistic’ and ‘not always rational’, underlining that the two groups do not always see eye‐to‐eye. This could lead to conflicting views on key issues such as independence and self‐determination which will be relevant when responding to the American interest. For instance, politicians may favour a quicker exit from the Kingdom of Denmark, seeing it as advantageous in the current political climate, whereas state elites—who are more closely tied to Danish institutional frameworks—may fear the administrative and economic instability such a move could bring, especially if it meant adopting systems less compatible with Greenland's state‐led growth model, as would be the case, in a U.S.‐style economy. With a potentially divided elite, decision‐making could be stalled by competing visions.

Furthermore, the potential for fragmentation between political elites and other elites becomes particularly problematic in the context of Greenland's state‐led power elite in which private business and state owned corporations have almost equal representation in the Greenlandic power elite. This makes the state both regulator and key market actor. While state administration elites can be seen as promoting their fraction in public business, politicians may use this to show how small business owners lose out. A case of this was seen in the recent general election, which partly reflected a voter backlash against a fisheries law from 2024, perceived to favour the strongly embedded large fishing enterprises at the expense of indigenous coastal small‐scale fishermen.^5^ Demokraatit were critical of the law during the campaign and gained significant support in part because of that stance, but after the election, they entered into a coalition government with Siumut, Atassut and Inuit Ataqatigiit and ultimately chose not to revise the law, thus ending up, seemingly, as a case of the state favouring state owned enterprises. This illustrates how the elite divisions and structure of the political economy of Greenland can lead to political tensions, reduced legitimacy, and uneven representation of economic interests in policymaking.

For now, the Greenlandic Government is rejecting American advances—perhaps because the political and administrative elite also base their power on distributing the annual subsidies from Copenhagen. If fractions of the political elite decides to side with American and turn the issue of Greenland's sovereignty into noisy political struggle, the question is if the rest of the Greenlandic elite can act cohesively—as the British business elite were incapable of during Brexit (Feldmann and Morgan 2021)—or if the sectoral and ethnic fracturing is too strong. As this triangular drama about geopolitical dominion over Greenland involves current NATO partners, the cohesiveness of the ties of the Greenlandic elite may have large implications for the European‐American relations during and following Trump's second term.

## Conflicts of Interest

The authors declare no conflicts of interest.

## Data Availability

The data that support the findings of this study are available on request from the corresponding author. The data are not publicly available due to privacy or ethical restrictions.

## Endnotes

^1^
For media coverage of these events, see for example https://www.bbc.com/news/articles/crkezj07rzro or https://www.bbc.com/news/articles/c5yv10knyd9o.

^2^
This is similar to for example American Samoa (0,42), but less than for example Bermuda (0.75) or the British Virgin Islands (0.50), but more than Falkland Islands (0.33) (Alberti and Goujon 2019).

^3^
See for example, https://www.zetland.dk/historie/sA9RuxNL‐aopXLwdn‐c3bb1.

^4^
The *k*‐circle algorithm operates in several stages. Initially, it examines which individuals have at least three posts, and which forums contain at least two of these so‐called ‘brokers’. All other individuals and forums are then excluded from the dataset. The remaining individuals and forums are assigned a *k*‐value of 2, indicating they act as brokers between at least three forums, each containing at least two brokers. The algorithm then repeats, but with the broker requirement increased by one, while maintaining the condition of three posts across the remaining forums. This iterative process peels away layers of the network, with each forum and individual being assigned a *k*‐value reflecting their position in the process. The final individuals and forums left will connect brokers with the most posts across multiple forums. For example, a forum with a k‐value of four indicates that it has at least four brokers, each with three posts in other forums with a similar number of brokers. In this way, the concept of power is translated into network terms, with brokers—those individuals embedded in significant networks—forming the core unit of analysis (Larsen and Ellersgaard 2017). The algorithm does not always identify one cohesive group, and it is not guaranteed that the most central part of the network will contain individuals formally holding powerful positions. However, it allows us to test whether a group emerges that behaves as a power elite, inline with Mills’ definition. We can then assess whether this group includes formally powerful individuals, allowing us to characterise the Greenlandic elite as a power elite.

^5^
See for example, https://www.berlingske.dk/politik/ny‐groenlandsk‐regering‐skuffer‐fiskere‐forudser‐kort‐levetid.
